# Binomial-discrete Erlang-truncated exponential mixture and its application in cancer disease

**DOI:** 10.1038/s41598-023-38709-2

**Published:** 2023-07-28

**Authors:** Alaa R. El-Alosey, Hussein Eledum

**Affiliations:** 1 Department of Mathematics, Faculty of Science, Tanta University, Tanta, 31527 Egypt; 2 Department of Statistics, Faculty of Science, University of Tabuk, Tabuk, Kingdom of Saudi Arabia

**Keywords:** Engineering, Mathematics and computing

## Abstract

Among diseases, cancer exhibits the fastest global spread, presenting a substantial challenge for patients, their families, and the communities they belong to. This paper is devoted to modeling such a disease as a special case. A newly proposed distribution called the binomial-discrete Erlang-truncated exponential (BDETE) is introduced. The BDETE is a mixture of binomial distribution with the number of trials (parameter $n$) taken after a discrete Erlang-truncated exponential distribution. A comprehensive mathematical treatment of the proposed distribution and expressions of its density, cumulative distribution function, survival function, failure rate function, Quantile function, moment generating function, Shannon entropy, order statistics, and stress-strength reliability, are provided. The distribution's parameters are estimated using the maximum likelihood method. Two real-world lifetime count data sets from the cancer disease, both of which are right-skewed and over-dispersed, are fitted using the proposed BDETE distribution to evaluate its efficacy and viability. We expect the findings to become standard works in probability theory and its related fields.

## Introduction

Cancer is the disease that spreads the most quickly around the world. It is a big problem for patients, their families, and their communities. If this sickness is caught early, it can be treated. Because of this, modelling of disease has become an important tool in the areas of public health research and disease epidemiology in the past few years.

A mixed distribution in statistics is the mixing of two or more probability distributions. It may be used to represent a statistical population with subpopulations, where the weights are the percentage of each subpopulation in the overall population and the mixture probability density components are the subpopulation densities. The probability distributions of the subpopulations may be univariate or multivariate and discrete or continuous. Also, the mixture distribution can come from different distribution families or the same distribution families with different parameters. Certain data sets may be suitable for a mixed distribution because discrete subgroups of the whole data set have unique characteristics that are best described independently.

In recent years, the challenge of creating a mixing distribution from the binomial distribution has gained a lot of attention. Breslow and Day^1^ extensively utilized negative binomial distribution in their cancer research statistics. Roy et al.^2^ investigated the Poisson mixture of the binomial distribution. Wood^3^ used a cumulative distribution function to come up with the mixture of the binomial distribution. Binomial mixes of the Poisson, normal, chi-squared, F, t, beta, gamma, exponential, rectangular, and Erlang distributions were developed by Roy et al.^4^. Zhu et al.^5^ recently used a beta-binomial-Poisson mixture distribution to model the number of successes and the number of binary trials at the same time. Shkedy et al.^6^ created the hierarchical Binomial-Poisson model, assuming that the number of responses is a Poisson random variable, for the analysis of a crossover design for correlated binary data when the number of trials depends on the dose. To predict how many credits first-year students at the University of Florence's School of Economics will obtain, Grilli et al.^7^ used a binomial finite mixture model. Knape et al.^8^ tested the sensitivity of binomial N-mixture models to over-dispersion in abundance and detection using simulations and a case study. El-Alosey^9^ proposed the binomial-exponential mixture by deriving the probability mass function of discrete mixes of distributions using the probability-generating function approach. The Erlang distribution's binomial mixture was created by Abed Al-Kadim and AL-Hussani^10^ utilizing moment's technique and Laplace transform.

Very recently, Triple Binomials, defined as a multiplicative mixing of the same three distributions, were developed by Adnan and Kiser^11^. Eledum and El-Alosey^12^ derived the binomial-geometric mixtures by using the probability-generating function technique.

Mixture distributions can be applied in cancer disease to identify different subtypes and stages of the disease based on the expression of biomarkers. This approach can lead to better diagnosis, prognosis, and treatment of cancer patients. Prabakaran et al.^13^ developed the Gaussian mixture model (GMM)-based classifier to improve molecular stratification of patients with breast cancer. Gaussian Mixture Models are often used for clustering and classification tasks in epidemiology. Their application in genotyping and disease subtyping has been explored in numerous studies, as highlighted by McLachlan et al.^14^. Held et al.^15^ applied the Beta distribution to infectious disease data analysis. Noor et al.^16^ preferred a novel four-component mixture model under Bayesian estimation to estimate the average number of incidences and deaths of both genders in different age groups, considering 28 different kinds of cancer diagnosed in recent years. In this paper, the proposed mixture distribution is fitted to two datasets of cancer disease, and the results showed that the proposed mixture distribution is well suited to model these datasets. In other words, we devote this paper to modeling a cancer disease using a new mixture distribution called the binomial-discrete Erlang-truncated exponential distribution (BDETE). This mixture distribution is a combination of the binomial distribution with the discrete Erlang-truncated exponential distribution. We use the probability-generating function of mixtures to find the pmf of the BDETE distribution. We look at some statistical properties of the proposed distribution and use the MLE to estimate its parameters.

The proposed BDETE distribution with three parameters is interesting because it has an increasing hazard rate function and a decreasing probability mass function. The novel lifetime mixture distribution is useful because it can model a real lifetime count data set of cancer disease that is skewed to the right and over-dispersed.

### Binomial and discrete Erlang-truncated exponential distributions

The probability mass function (pmf) and the associated probability-generating function (pgf) of a binomial random variable $X$ with parameters $n$ and $p$ are given as

1 $${f}_{X}\left(x;n,p\right)=\left(\begin{array}{c}n\\ x\end{array}\right){p}^{n} {(1-p)}^{n-x} ;x=\mathrm{0,1},2,\dots ,n, , 0\le p\le 1$$

2 $${P}_{X}\left(z;n,p\right)=E\left({Z}^{x}\right)= {\left[\left(1-p\right)+pz\right]}^{n}$$

The pmf of a discrete Erlang-truncated exponential (DETE) random variable $N$ with parameters $n$, $\beta$ and $\omega$ is given as^17^

3 $${f}_{N}\left(n; \beta ,\omega \right)={\omega }^{\beta n}\left(1-{\omega }^{\beta }\right) , n\in \left\{\mathrm{0,1},2,\dots \right\}, \beta >0, 0<\omega <1$$

where $n$ is the number of failures before the first success. The DETE distribution's mean and variance are stated as

$${\mu }_{N}=1/(1-{\omega }^{\beta })$$

$${\sigma }_{N}^{2}={\omega }^{\beta }/{(1-{\omega }^{\beta })}^{2}$$

### Mixing binomial and other distributions with a probability-generating function method

If we assume that the parameter $n$ in the binomial distribution in Eq. (1) is a random variable with pmf ${f}_{N}(n,\omega ,p)$, then we can use the probability generating function approach to get the binomial mixed distribution as^12^

4 $${P}_{D}\left(d; p,\omega ,\beta \right)=\sum_{n=0}^{\infty }{{P}_{D}\left(d;n,p\right)f}_{N}(n,\omega ,\beta )$$

where ${P}_{D}(d;n,p)$ is pgf for the binomial distribution, while $p$, $\omega$ and $\beta$ are the parameters of the mixture distribution.

This paper's remaining sections are organized as follows: The proposed distribution BDETE is presented in “Binomial-discrete Erlang-truncated exponential distribution” section, and “Distributional properties” section demonstrates its statistical features, including the quantile function, the moment-generating function, the Shannon entropy, the order statistics, and the stress-strength parameter. The maximum likelihood technique is described in “Maximum likelihood estimation” section for estimating BDETE mixing parameters. In “Application” section, two real data sets are used to illustrate the performance of the BDETE distribution. Finally, some final thoughts are offered in “Conclusion remarks” section.

## Binomial-discrete Erlang-truncated exponential distribution

This section evaluates and discusses the mathematical formulae for the pmf and cdf of the proposed Binomial-discrete Erlang-truncated exponential mixture (BDETE**)**. Here we also derive the hazard and survival functions for the BDETE distribution.

### Probability mass and cumulative distribution functions for the BDETE

If we assume that the parameter $n$ in the binomial distribution in Eq. (1) follows a discrete Erlang-truncated exponential distribution in Eq. (4), we can use the probability generating function method in Eq. (2) to get the pmf of the proposed BDETE distribution as

$$\begin{aligned} {P}_{D}\left(d;\mathrm{ p}, \omega ,\upbeta \right) & =\sum_{n=0}^{\infty }{{P}_{D}\left(d;n,p\right)f}_{N}(n; \omega ,\upbeta ) \\ & =(1-{\omega }^{\beta })\sum_{n=0}^{\infty }{\left[q{\omega }^{\beta }+{\omega }^{\beta }pd\right]}^{n}; q=(1-p) \\ &=\frac{1-{\omega }^{\beta }}{1-(1-p){\omega }^{\beta }-p{\omega }^{\beta }d} \\ &=\frac{1-{\omega }^{\beta }}{\left[1-(1-p){\omega }^{\beta }\right]\left[1-\frac{p{\omega }^{\beta }d}{1-(1-p){\omega }^{\beta }}\right]} \\ &=\frac{1-{\omega }^{\beta }}{1-(1-p){\omega }^{\beta }}\sum_{i=0}^{\infty }{\left[\frac{p{\omega }^{\beta }d}{1-(1-p){\omega }^{\beta }}\right]}^{i} \end{aligned}$$

Thus, the pmf of BDETE is the coefficient of ${d}^{x}$ in the pgf as

5 $$f\left(x;p,\omega ,\beta \right)=\frac{{\omega }^{\beta x} {p}^{x}\left(1-{\omega }^{\beta }\right)}{{\left[1-{\omega }^{\beta }(1-p)\right]}^{x+1}} , x=\mathrm{0,1},2,\dots , 0\le p\&\omega \le 1, \beta >0$$

with the corresponding cdf as:

6 $${F}_{X}\left(x; p, \omega ,\beta \right)=1-{\left[\frac{{\omega }^{\beta }p}{1-{\omega }^{\beta }\left(1-p\right)}\right]}^{x+1}$$

where $$x\in \left\{\mathrm{0,1},2,\dots \right\}, 0\le p\&\omega \le 1,\beta >0$$

The binomial-geometric distribution can be obtained from Eq. (5) by taking $\beta =1$ and $\omega =1-\theta$ as follows^12^

$$f\left(x;p,\theta \right)=\frac{\theta {p}^{x}{\left(1-\theta \right)}^{x}}{{\left[1-(1-p)\left(1-\theta \right)\right]}^{x+1}} x=\mathrm{0,1},2,\dots , 0\le p\&\theta \le 1$$

**Figure 1:**
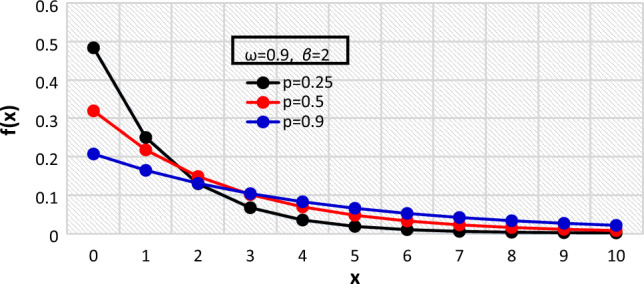
The ${f}_{X}\left(x\right)$ of the BDETE at $p=0.25, 0.5, 0.9$ when $\omega =0.9 \& \beta =2$.

**Figure 2:**
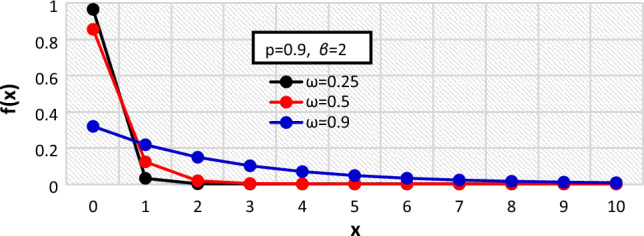
The ${f}_{X}\left(x\right)$ of the BDETE at $\omega =0.25, 0.5, 0.9$ when $p=0.9 \& \beta =2$.

**Figure 3:**
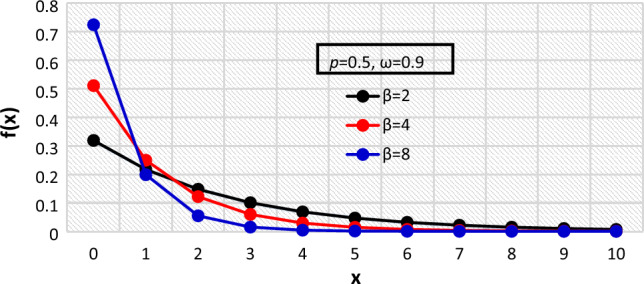
The ${f}_{X}\left(x\right)$ of the BDETE at $\beta =\mathrm{2,4},8$ when $p=0.5 \& \omega =0.9$.

**Figure 4:**
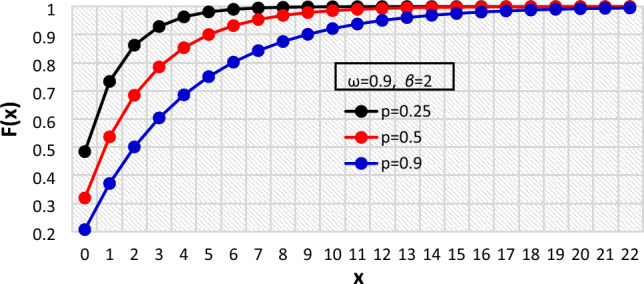
The ${F}_{X}\left(x\right)$ of the BDETE at $p=0.25, 0.5, 0.9$ when $\omega =0.9 \& \beta =2$.

**Figure 5:**
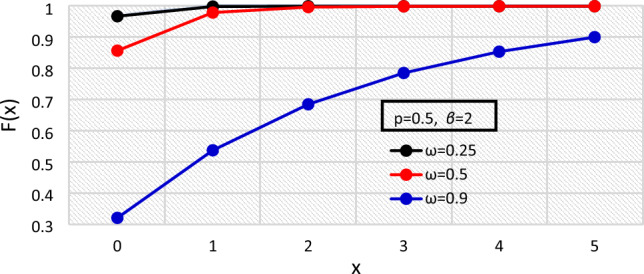
The ${F}_{X}\left(x\right)$ of of the BDETE at $\omega =0.25, 0.5, 0.9$ when $p=0.5 \& \beta =2$.

**Figure 6:**
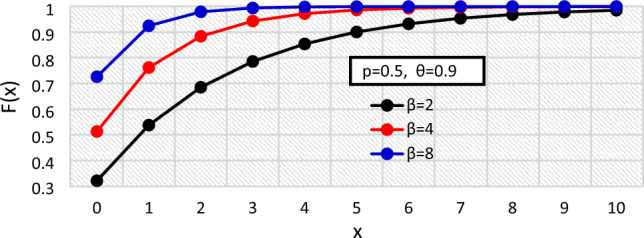
The ${F}_{X}\left(x\right)$ of the BDETE at $\beta =\mathrm{2,4},8$ when $p=0.5 \& \omega =0.9$.

The pmf of the BDETE distribution for varying values of the distribution’s parameters are shown in Figs. 1, 2, and 3, while the cdf are presented in Figs. 4, 5 and 6.

The proposed BDETE distribution is right-skewed, and its pmf is a declining function, as shown in Figs. 1 through 3.

### Survival and hazard rate functions

The survival function of X is:

7 $${S}_{X}\left(x; p, \omega ,\beta \right)=1-{F}_{X}\left(x-1; p, \omega ,\upbeta \right)={\left[\frac{{\omega }^{\beta } p}{1-{\omega }^{\beta } (1-p)}\right]}^{x}$$

The hazard function is as follows:

$${H}_{X}\left(x; p,\omega , \beta \right)=\frac{f\left(x; p, \omega , \beta \right)}{{S}_{X}\left(x; p, \omega , \beta \right)}=\frac{1-{ \omega }^{\beta }}{1-{ \omega }^{\beta }(1-p)}$$

**Figure 7:**
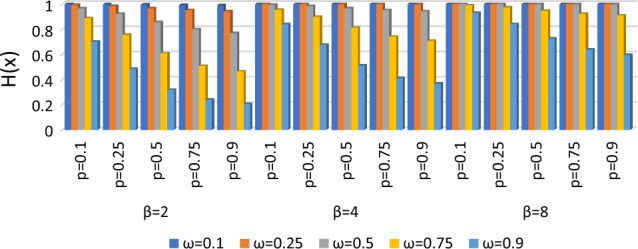
The *H(x)* of the BDETE combination values of $p, \omega$ and $\beta$.

The hazard function of the BDETE is shown in Table 1 and Fig. 7 for a given set of $p, \omega$ and $\beta$ values.

**Table 1:** Hazard function of BDETE distribution for combination values of $p, \omega$ and $\beta$.

		$\omega$=0.1	$\omega$=0.25	$\omega$=0.5	$\omega$=0.75	$\omega$=0.9
$\beta =2$	$p=$ 0.1	0.9990	0.9934	0.9677	0.8861	0.7011
	$p=$ 0.25	0.9975	0.9836	0.9231	0.7568	0.4841
	$p=$ 0.5	0.9950	0.9677	0.8571	0.6087	0.3193
	$p=$ 0.75	0.9925	0.9524	0.8000	0.5091	0.2382
	$p=$ 0.9	0.9910	0.9434	0.7692	0.4636	0.2067
$\beta =4$	$p=$ 0.1	1.0000	0.9996	0.9934	0.9558	0.8398
	$p=$ 0.25	1.0000	0.9990	0.9836	0.8963	0.6771
	$p=$ 0.5	0.9999	0.9980	0.9677	0.8121	0.5118
	$p=$ 0.75	0.9999	0.9971	0.9524	0.7423	0.4114
	$p=$ 0.9	0.9999	0.9965	0.9434	0.7059	0.3680
$\beta =8$	$p=$ 0.1	1.0000	1.0000	0.9996	0.9890	0.9297
	$p=$ 0.25	1.0000	1.0000	0.9990	0.9729	0.8411
	$p=$ 0.5	1.0000	1.0000	0.9980	0.9473	0.7257
	$p=$ 0.75	1.0000	1.0000	0.9971	0.9230	0.6382
	$p=$ 0.9	1.0000	1.0000	0.9965	0.9090	0.5952

Based on Table 1 and Fig. 7, we observe that the hazard function goes down as both $p$ and $\theta$ go up. On the other hand, as $\beta$ goes up, the hazard function goes up.

## Distributional properties

In this section, we develop some statistical properties of the BDETE distribution, such as the quantile function, the moment generating function, and some other related measures. We also define some other techniques, like the Shannon entropy and the order statistics.

### Quantile function

By inverting the cdf in Eq. (6), the quantile of order $0<r<1$ could be derived as follows

$${\mathrm{F}}_{\mathrm{X}}\left(\mathrm{Q};\upomega ,\upbeta \right)=1-{\left[\frac{{\omega }^{\beta } p}{1-{\omega }^{\beta } (1-p)}\right]}^{\mathrm{Q}+1}$$

Then $${\mathrm{F}}_{\mathrm{X}}^{-1}\left(\mathrm{r}\right)=\mathrm{min}\{\mathrm{x}\in \mathrm{R}:{\mathrm{F}}_{\mathrm{X}}(\mathrm{x})\ge \mathrm{r}\}$$

$$1-{\left[\frac{{\omega }^{\beta } p}{1-{\omega }^{\beta } (1-p)}\right]}^{\mathrm{Q}+1}=\mathrm{r}$$

Thus, The ${r}^{th}$ quantile is

8 $$\mathrm{Q}\left(r;\mathrm{ p},\upomega \right)=\frac{{\mathrm{log}}_{2}\left( 1-r\right)}{{\mathrm{log}}_{2}\left( {\omega }^{\beta }p\right)-{\mathrm{log}}_{2}\left[ 1-{\omega }^{\beta }(1- p)\right] }-1$$

The BDETE distribution's median can be computed by substituting by $r=\frac{1}{2}$ in Eq. (8) as follows:

$${\mathrm{Q }}_{0.5}=\mathrm{Q}\left(\gamma ;\mathrm{p},\upomega \right)=\frac{-1}{{\mathrm{log}}_{2}\left( {\omega }^{\beta }p\right)-{\mathrm{log}}_{2}\left[ 1-{\omega }^{\beta }(1- p)\right]}-1$$

### The moment-generating function

The moment-generating function of a random variable $X$ with a BDETE and parameters $(p,\omega ,\beta )$ is deduced as

$${M}_{X}\left(t \right)=E({e}^{tx})$$

9 $$=\frac{1-{\omega }^{\beta }}{1-{\omega }^{\beta }\left(1-p\right)-p {\omega }^{\beta }{e}^{t}}$$

The mean (first moment) of the BDETE distribution can be calculated using Eq. (9) as follows:

10 $${\mu }_{1}=\mu =E\left(X\right)={\left.\frac{d{M}_{X}\left(t \right)}{dt}\right|}_{t=0}=\frac{{\omega }^{\beta }p}{1-{\omega }^{\beta }}$$

The 2nd moment about the origin is

$${\mu }_{2}=E\left({X}^{2}\right) =\frac{{\omega }^{\beta }p}{1-{\omega }^{\beta }}+\frac{2{{\omega }^{2\beta }p}^{2}}{{(1-{\omega }^{\beta })}^{2}}$$

As a result, the BDETE distribution's variance is given by

11 $${\sigma }^{2}={\mu }_{2}-{\mu }_{1}^{2}=\frac{{\omega }^{\beta }p}{1-{\omega }^{\beta }}+\frac{{{\omega }^{2\beta }p}^{2}}{{\left(1-{\omega }^{\beta }\right)}^{2}}$$

It is obvious from Eqs. (10) and (11) that

$${\sigma }^{2}=\mu +{\mu }^{2}>\mu$$

This demonstrates that the BDETE distribution is always over-dispersed (the variance is larger than the mean), making it appropriate for usage with such data.

The 3rd moment about the origin is

$${\mu }_{3}=E\left({X}^{3}\right)=\frac{{\omega }^{\beta }p}{1-{\omega }^{\beta }}+\frac{{6{\omega }^{2\beta }p}^{2}}{{(1-{\omega }^{\beta })}^{2}}+\frac{{6{\omega }^{3\beta }p}^{3}}{{(1-{\omega }^{\beta })}^{3}}$$

The 4th moment about the origin is

$${\mu }_{4}=E\left({X}^{4}\right)=\frac{{\omega }^{\beta }p}{1-{\omega }^{\beta }}+\frac{{14 {\omega }^{2\beta }p}^{2}}{{(1-{\omega }^{\beta })}^{2}}+\frac{{36 {\omega }^{3\beta }p}^{3}}{{(1-{\omega }^{\beta })}^{3}}+\frac{{24 {\omega }^{4\beta }p}^{4}}{{(1-{\omega }^{\beta })}^{4}}$$

The BDETE distribution has a coefficient of variation (C.V), coefficient of Skewness ($\sqrt{{\beta }_{1}}$), the coefficient of Kurtosis (${\beta }_{2}$), and the index of dispersion ($\gamma$) as

$$C.V=\frac{\sigma }{{\mu }_{1}}=\sqrt{\frac{1-{\omega }^{\beta }(1-p)}{{\omega }^{\beta }p}}$$

$$\sqrt{{\beta }_{1}}=\frac{{\mu }_{3}-3\mu {\sigma }^{2}-{\mu }^{3}}{{\left({\sigma }^{2}\right)}^\frac{3}{2}} =\left[\frac{{\omega }^{\beta }p}{1-{\omega }^{\beta }}+\frac{{3{ \omega }^{2\beta }p}^{2}}{{(1-{\omega }^{\beta })}^{2}}+\frac{{2{\omega }^{3\beta }p}^{3}}{{(1-{\omega }^{\beta })}^{3}}\right]\div {\left[\frac{{\omega }^{\beta }p}{1-{\theta \omega }^{\beta }}+\frac{{{\omega }^{2\beta }p}^{2}}{{(1-{\omega }^{\beta })}^{2}}\right]}^\frac{3}{2}$$

$${\beta }_{2}=\frac{{\mu }_{4}-4\mu {\mu }_{3}+6{\mu }_{2}{\mu }^{2}-3{\mu }^{4}}{{\left({\sigma }^{2}\right)}^{2}} =\frac{\left[1+\frac{10\left({\omega }^{\beta }p\right)}{1-{\omega }^{\beta }}+\frac{{18{\omega }^{2\beta }p}^{2}}{{\left(1-{\omega }^{\beta }\right)}^{2}}+\frac{{48\left({\omega }^{\beta }p\right)}^{3}}{{\left(1-{\omega }^{\beta }\right)}^{3}}\right]}{\left[\frac{{\omega }^{\beta }p}{1-{\omega }^{\beta }}+\frac{{2{\omega }^{2\beta }p}^{2}}{{(1-{\omega }^{\beta })}^{2}}+\frac{{{\omega }^{3\beta }p}^{3}}{{(1-{\omega }^{\beta })}^{3}}\right]}$$

$$\gamma =\frac{{\sigma }^{2}}{{\mu }_{1}}=\frac{1-(1-p){\omega }^{\beta }}{1-{\omega }^{\beta }}$$

Table 2 shows the mean, variance, and skewness of the BDETE distribution for various combinations of $p, \omega$ and $\beta$.

**Table 2:** Mean, variance, and skewness of BDETE for different values of distribution’s parameters.

$\beta$	$p$		$\omega$=0.1	$\omega$=0.25	$\omega$=0.5	$\omega$=0.75	$\omega$=0.9
2	0.1	$\upmu$	0.0010	0.0067	0.0333	0.1286	0.4263
		${\sigma }^{2}$	0.0010	0.0067	0.0344	0.1451	0.6081
		$\sqrt{{\beta }_{1}}$	31.5119	12.3696	5.7474	3.3003	2.3758
	0.25	$\upmu$	0.0025	0.0167	0.0833	0.3214	1.0658
		${\sigma }^{2}$	0.0025	0.0169	0.0903	0.4247	2.2017
		$\sqrt{{\beta }_{1}}$	19.9750	7.9383	3.8829	2.5208	2.1105
	0.5	$\upmu$	0.0051	0.0333	0.1667	0.6429	2.1316
		${\sigma }^{2}$	0.0051	0.0344	0.1944	1.0561	6.6752
		$\sqrt{{\beta }_{1}}$	14.1776	5.7474	3.0237	2.2242	2.0371
	0.75	$\upmu$	0.0076	0.0500	0.2500	0.9643	3.1974
		${\sigma }^{2}$	0.0076	0.0525	0.3125	1.8941	13.4205
		$\sqrt{{\beta }_{1}}$	11.6193	4.8008	2.6833	2.1279	2.0185
	0.9	$\upmu$	0.0091	0.0600	0.3000	1.1571	3.8368
		${\sigma }^{2}$	0.0092	0.0636	0.3900	2.4961	18.5582
		$\sqrt{{\beta }_{1}}$	10.6306	4.4411	2.5621	2.0978	2.0134
4	0.1	$\upmu$	0.0000	0.0004	0.0067	0.0463	0.1908
		${\sigma }^{2}$	0.0000	0.0004	0.0067	0.0484	0.2272
		$\sqrt{{\beta }_{1}}$	316.2167	50.5272	12.3696	4.9648	2.8986
	0.25	$\upmu$	0.0000	0.0010	0.0167	0.1157	0.4770
		${\sigma }^{2}$	0.0000	0.0010	0.0169	0.1291	0.7044
		$\sqrt{{\beta }_{1}}$	199.9975	31.9844	7.9383	3.4272	2.3280
	0.5	$\upmu$	0.0001	0.0020	0.0333	0.2314	0.9539
		${\sigma }^{2}$	0.0001	0.0020	0.0344	0.2850	1.8639
		$\sqrt{{\beta }_{1}}$	141.4249	22.6495	5.7474	2.7402	2.1299
	0.75	$\upmu$	0.0001	0.0029	0.0500	0.3471	1.4309
		${\sigma }^{2}$	0.0001	0.0029	0.0525	0.4677	3.4782
		$\sqrt{{\beta }_{1}}$	115.4773	18.5203	4.8008	2.4776	2.0706
	0.9	$\upmu$	0.0001	0.0035	0.0600	0.4166	1.7170
		${\sigma }^{2}$	0.0001	0.0035	0.0636	0.5901	4.6653
		$\sqrt{{\beta }_{1}}$	105.4182	16.9215	4.4411	2.3863	2.0529
8	0.1	$\upmu$	0.0000	0.0000	0.0004	0.0111	0.0756
		${\sigma }^{2}$	0.0000	0.0000	0.0004	0.0112	0.0813
		$\sqrt{{\beta }_{1}}$	31,622.78	809.5388	50.5272	9.6384	4.0374
	0.25	$\upmu$	0.0000	0.0000	0.0010	0.0278	0.1890
		${\sigma }^{2}$	0.0000	0.0000	0.0010	0.0286	0.2247
		$\sqrt{{\beta }_{1}}$	20,000	511.9990	31.9844	6.2436	2.9071
	0.5	$\upmu$	0.0000	0.0000	0.0020	0.0556	0.3779
		${\sigma }^{2}$	0.0000	0.0000	0.0020	0.0587	0.5207
		$\sqrt{{\beta }_{1}}$	14,142.14	362.0401	22.6495	4.5859	2.4332
	0.75	$\upmu$	0.0000	0.0000	0.0029	0.0834	0.5669
		${\sigma }^{2}$	0.0000	0.0000	0.0029	0.0904	0.8882
		$\sqrt{{\beta }_{1}}$	11,547.01	295.6062	18.5203	3.8810	2.2640
	0.9	$\upmu$	0.0000	0.0000	0.0035	0.1001	0.6802
		${\sigma }^{2}$	0.0000	0.0000	0.0035	0.1102	1.1430
		$\sqrt{{\beta }_{1}}$	10,540.93	269.85	16.9215	3.6164	2.2079

The results in Table 2 show that when both $p$ and $\omega$ increase, so do the proposed distribution's mean and variance. Conversely, when $\beta$ rises, the mean and variance fall. On the other hand, when both $p$ and $\omega$ increase, the coefficient of skewness decreases, while when $\beta$ rises, so do the coefficient of skewness. Table 2 also demonstrates that the proposed BDETE distribution has over-dispersion and positive skewness.

### Shannon entropy

The Shannon entropy is one of many entropy and information indices that have been made and used in a wide range of fields and situations. This measure is defined as

$$H\left(X\right)=E\left\{-\mathrm{log}\left[f\left(x\right)\right]\right\}$$

The Shannon entropy of a random variable $X$ with a BDETE distribution pmf of Eq. (5) is

$$\begin{aligned} H\left(X\right) & =-\sum_{x=0}^{\infty }f\left(x\right) \mathrm{log}\left[f\left(x\right)\right]\\ &=\mathrm{log}\left[\frac{1-{\omega }^{\beta }\left(1-p\right)}{1-{\omega }^{\beta }}\right]-\frac{{\omega }^{\beta } p}{1-{\omega }^{\beta }\left(1-p\right)}\mathrm{log}\left[\frac{{\omega }^{\beta } p}{1-{\omega }^{\beta }(1-p)}\right] \end{aligned}$$

### Order statistics

In the field of non-parametric statistics and inference, order statistics are the most significant and fundamental tools. They employ a variety of approaches to address estimation and hypothesis testing issues. Therefore, the purpose of this subsection is to develop some order statistics for the BDETE distribution, including the maximum, minimum, and median order statistics.

Suppose ${f}_{k}(x;p, \omega ,\beta )$ and ${F}_{k}(x;p, \omega ,\beta )$ are the pmf and cdf of the kth order statistic of a random sample; ${X}_{1},{X}_{2},\dots ,{X}_{n}$*;* of size $\mathrm{n}$, taken from BDETE.

The kth order statistic's pmf is

$$\begin{aligned} {f}_{k}\left(x;p ,\omega ,\beta \right) &=\frac{n!}{\left(k-1\right)!\left(n-k\right)!}{\left[F\left(x;p, \omega ,\beta \right)\right]}^{k-1}{\left[1-F\left(x;p, \omega ,\beta \right)\right]}^{n-k}f\left(x;p, \omega ,\beta \right) \\ &= \frac{n!}{\left(k-1\right)!\left(n-k\right)!}\sum_{j=0}^{k-1}{\left(-1\right)}^{j}\left(\genfrac{}{}{0pt}{}{k-1}{j}\right)\frac{1-{\omega }^{\beta }}{1-{\omega }^{\beta }(1-p)}{\left(\frac{{\omega }^{\beta } p}{1-{\omega }^{\beta }(1-p)}\right)}^{x\left(n-k+j+1\right)+n+j-k} \end{aligned}$$

The kth order statistic's cdf is

$$\begin{aligned}{F}_{k}\left(x;p, \omega ,\beta \right) & =\sum_{i=k}^{n}\left(\genfrac{}{}{0pt}{}{n}{i}\right){\left[F\left(x;\lambda ,\omega , \beta \right)\right]}^{i}{\left[1-F\left(x;\lambda ,\omega , \beta \right)\right]}^{n-i} \\ &=\sum_{i=k}^{n}\sum_{j=0}^{n}{\left(-1\right)}^{j}\left(\genfrac{}{}{0pt}{}{n}{i}\right)\left(\genfrac{}{}{0pt}{}{n}{j}\right){\left(\frac{{\omega }^{\beta } p}{1-{\omega }^{\beta }(1-p)}\right)}^{(x+1)(n-i+j)}\end{aligned}$$

Let ${X}_{(1)}=min({X}_{1},{X}_{2},\dots ,{X}_{n})$*,*${X}_{(n)}=max({X}_{1},{X}_{2},\dots ,{X}_{n})$*, and* ${X}_{(m+1)}$ with $\mathrm{m}=\frac{\mathrm{n}}{2}$ be the minimum, maximum and medium order statistics, respectively. Therefore, result, the pmfs of the minimum, maximum, and median are

$${f}_{1}\left(x;p,\omega , \beta \right)= \frac{n(1-{\omega }^{\beta })}{1-{\omega }^{\beta }(1-p)}{\left(\frac{{\omega }^{\beta } p}{1-{\omega }^{\beta }(1-p)}\right)}^{n\left(x+1\right)-1}$$

$${f}_{n}\left(x;p,\omega , \beta \right)=\frac{n(1-{\omega }^{\beta }) }{1-{\omega }^{\beta }(1-p)} {\left(\frac{{\omega }^{\beta } p}{1-{\omega }^{\beta }(1-p)}\right)}^{x}{\left[1-{\left(\frac{{\omega }^{\beta } p}{1-{\omega }^{\beta }(1-p)}\right)}^{(x+1)}\right]}^{n-1}$$

$${f}_{m+1}\left(x;p, \omega ,\beta \right)= \frac{n!}{\left(m\right)!\left(n-m+1\right)!}\frac{n(1-{\omega }^{\beta }) }{1-{\omega }^{\beta }(1-p)}{\left(\frac{{\omega }^{\beta } p}{1-{\omega }^{\beta }(1-p)}\right)}^{\left(n-m\right)\left(x+1\right)-1}{\times \left[1-{\left(\frac{{\omega }^{\beta } p}{1-{\omega }^{\beta }(1-p)}\right)}^{(x+1)}\right]}^{m}$$

### Estimation of Stress-strength for BDETE distribution

In this part, we look at how to estimate the stress-strength parameter when both the strength and the stress are random variables with the BDETE distribution.

The discrete version of a stress-strength parameter is specified as

$$\mathrm{R}=\mathrm{P}\left(\mathrm{X}>\mathrm{Y}\right)=\sum_{\mathrm{x}=0}^{\infty }{\mathrm{f}}_{\mathrm{X}}(\mathrm{x}){\mathrm{F}}_{\mathrm{Y}}(\mathrm{x})$$

where ${\mathrm{f}}_{\mathrm{X}}(\mathrm{x})$ and ${\mathrm{F}}_{\mathrm{X}}(\mathrm{x})$ are the pmf and cdf of the independent discrete random variables X and Y, respectively.

Suppose X and Y are two independent random variables having the BDETE distribution with parameters BDETE(${\mathrm{p}}_{1}, {\upomega }_{1},{\upbeta }_{1}$) and BDETE(${\mathrm{p}}_{2}, {\upomega }_{2},{\upbeta }_{2}$) respectively. The stress-strength parameter for the BDETE distribution is given as

$$\begin{aligned} \mathrm{R} & =\sum_{\mathrm{x}=0}^{\infty }\left[\frac{(1-{\upomega }_{1}^{{\upbeta }_{1}}) }{1-(1-{\mathrm{p}}_{1}){\upomega }_{1}^{{\upbeta }_{1}}}\right]{\left[\frac{{\mathrm{p}}_{1}{\upomega }_{1}^{{\upbeta }_{1}}}{1-(1-{\mathrm{p}}_{1}){\upomega }_{1}^{{\upbeta }_{1}}}\right]}^{\mathrm{x}}\left\{1-{\left[\frac{{\mathrm{p}}_{2}{\upomega }_{2}^{{\upbeta }_{2}}}{1-(1-{\mathrm{p}}_{2}){\upomega }_{2}^{{\upbeta }_{2}}}\right]}^{\mathrm{x}+1}\right\} \\ &=1-\frac{(1-{\uptheta }_{1}^{{\upbeta }_{1}}){\mathrm{p}}_{2}{\uptheta }_{2}^{{\upbeta }_{2}} }{\left[1-(1-{\mathrm{p}}_{1}){\upomega }_{1}^{{\upbeta }_{1}}\right]\left[1-(1-{\mathrm{p}}_{2}){\upomega }_{2}^{{\upbeta }_{2}}\right]-{\mathrm{p}}_{1}{\mathrm{p}}_{2}{\upomega }_{1}^{{\upbeta }_{1}}{\upomega }_{2}^{{\upbeta }_{2}}}\end{aligned}$$

## Maximum likelihood estimation

The goal of this section is to find the maximum likelihood estimate (MLE) for the BDETE distribution parameters.

Let ${X}_{1},{X}_{2},\dots ,{X}_{n}$ be a random sample of size $n$ having the BDETE distribution. The log-likelihood is

12 $$\begin{aligned} \mathcalligra{l} & =n\mathrm{log}\left[\frac{1-{\omega }^{\beta }}{1-{\omega }^{\beta }\left(1-p\right)}\right]+\sum_{i=1}^{n}{x}_{i}\mathrm{log}\left[\frac{{\omega }^{\beta } p}{1-{\omega }^{\beta }\left(1-p\right)}\right] \\ &=n\mathrm{log}\left[1-{\omega }^{\beta }\right]-\left(\sum_{i=1}^{n}{x}_{i}+n \right)\mathrm{log}\left[1-{\omega }^{\beta }\left(1-p\right)\right]+\mathrm{log}\left({\omega }^{\beta } p\right) \sum_{i=1}^{n}{x}_{i} \end{aligned}$$

Further differentiating the log-likelihood in Eq. (12) partially with respect to $p$, $\omega$ and $\beta$, we get the likelihood equations as

13 $$\frac{\partial \mathcalligra{l}}{\partial \mathrm{p}}=\frac{\sum_{\mathrm{i}=1}^{\mathrm{n}}{\mathrm{x}}_{\mathrm{i}}}{\mathrm{p}}-\frac{{\upomega }^{\upbeta }\left(\sum_{\mathrm{i}=1}^{\mathrm{n}}{\mathrm{x}}_{\mathrm{i}}+\mathrm{n}\right)}{1-{\upomega }^{\upbeta }\left(1-\mathrm{p}\right)}=0$$

14 $$\frac{\partial \mathcalligra{l}}{\partial\upomega }=\frac{\upbeta \sum_{\mathrm{i}=1}^{\mathrm{n}}{\mathrm{x}}_{\mathrm{i}}}{\upomega }-\frac{\mathrm{n\beta }{\upomega }^{\upbeta -1}}{1-{\upomega }^{\upbeta }}-\frac{\left(1-\mathrm{p}\right)\left(\sum_{\mathrm{i}=1}^{\mathrm{n}}{\mathrm{x}}_{\mathrm{i}}+\mathrm{n}\right)\upbeta {\upomega }^{\upbeta -1}}{1-{\upomega }^{\upbeta }\left(1-\mathrm{p}\right)}=0$$

15 $$\frac{\partial \mathcalligra{l}}{\partial\upbeta }=\sum_{\mathrm{i}=1}^{\mathrm{n}}{\mathrm{x}}_{\mathrm{i}}\mathrm{log}[\upomega ]-\frac{\mathrm{n}{\upomega }^{\upbeta }\mathrm{log}\left[\upomega \right]}{1-{\upomega }^{\upbeta }}-\frac{\left(1-\mathrm{p}\right)\left(\sum_{\mathrm{i}=1}^{\mathrm{n}}{\mathrm{x}}_{\mathrm{i}}+\mathrm{n}\right){\upomega }^{\upbeta }\mathrm{log}\left[\upomega \right]}{1-{\upomega }^{\upbeta }\left(1-\mathrm{p}\right)}=0$$

The solutions of likelihood Eqs. (13), (14), and (15) provide the MLEs of $p$, $\omega$ and $\beta$, which can be obtained by numerical methods. Since the MLE of the vector of unknown parameters $\tau ={( p , \omega ,\upbeta )}^{T}$ cannot be derived in closed forms, it is, therefore, hard to figure out the exact MLEs for the BDETE’s parameters.

The second partial derivatives are given below

$$\frac{{\partial }^{2}\mathcalligra{l}}{\partial {p}^{2}}=\frac{{\upomega }^{2\beta }(\sum_{i=1}^{n}{x}_{i}+n)}{{\left[1-{\upomega }^{\beta }(1-p)\right]}^{2}}-\frac{\sum_{i=1}^{n}{x}_{i}}{{p}^{2}}$$

$$\frac{{\partial }^{2}\mathcalligra{l}}{\partial {\upomega }^{2}}=\frac{{(1-p)}^{2}(\sum_{i=1}^{n}{x}_{i}+n){{\beta }^{2}\upomega }^{2\beta -2}}{{\left[1-{\upomega }^{\beta }(1-p)\right]}^{2}}+\frac{(1-p)(\sum_{i=1}^{n}{x}_{i}+n){\beta (1-\beta )\upomega }^{\beta -2}}{\left[1-{\upomega }^{\beta }(1-p)\right]}-\frac{\beta \sum_{i=1}^{n}{x}_{i}}{{\upomega }^{2}}-\frac{n{{\beta }^{2}\upomega }^{2\beta -2}}{{\left[1-{\upomega }^{\beta }\right]}^{2}}-\frac{n{\beta (1-\beta )\upomega }^{\beta -2}}{1-{\upomega }^{\beta }}$$

$$\frac{{\partial }^{2}\mathcalligra{l}}{\partial {\beta }^{2}}={[\mathrm{log}(\upomega )]}^{2}{\upomega }^{\beta }\left\{\frac{-n}{{\left(1-{\upomega }^{\beta }\right)}^{2}}+\frac{(1-p)(\sum_{i=1}^{n}{x}_{i}+n)}{{\left[1-{\upomega }^{\beta }(1-p)\right]}^{2}}\right\}$$

$$\frac{{\partial }^{2}\mathcalligra{l}}{\partial p\partial\upomega }=\frac{(\sum_{i=1}^{n}{x}_{i}+n)\beta {\upomega }^{\beta -1}}{{\left[1-{\upomega }^{\beta }(1-p)\right]}^{2}}$$

$$\frac{{\partial }^{2}\mathcalligra{l}}{\partial p\partial \beta }=\frac{-(\sum_{i=1}^{n}{x}_{i}+n){\upomega }^{\beta }\mathrm{log}[\upomega ]}{{\left[1-{\upomega }^{\beta }(1-p)\right]}^{2}}$$

$$\frac{{\partial }^{2}l}{\partial\upomega \partial \beta }=\frac{\sum_{i=1}^{n}{x}_{i}}{\upomega }-\frac{n{\upomega }^{\beta -1}\left[1-\beta \mathrm{log}\left(\upomega \right)\right]}{1-{\upomega }^{\beta }}-\frac{n{\beta\upomega }^{2\beta -1}\mathrm{log}\left(\upomega \right)}{{\left(1-{\upomega }^{\beta }\right)}^{2}}+\frac{(1-p)(\sum_{i=1}^{n}{x}_{i}+n){\beta\upomega }^{\beta -1}\mathrm{log}(\upomega )}{{\left[1-{\upomega }^{\beta }(1-p)\right]}^{2}}$$

Lawless^18^ defined the asymptotic distribution of the MLE $\widehat{\tau }$ as

$$\left(\widehat{\tau }-\tau \right)\to N\left(0, {I}^{-1}\left(\tau \right)\right)$$

where ${I}^{-1}\left(\tau \right)$ is the inverse of Fisher’s information matrix of the unknown parameters $\tau ={( p ,\omega ,\beta )}^{T}$ as follows:

$${I}_{Y(p,\omega ,\beta )}\left(\tau \right)=\left[\begin{array}{ccc}-E\left(\frac{{\partial }^{2}\mathcalligra{l}}{\partial {p}^{2}}\right)& -E\left(\frac{{\partial }^{2}\mathcalligra{l}}{\partial p\partial \omega }\right)& -E\left(\frac{{\partial }^{2}\mathcalligra{l}}{\partial p\partial \beta }\right)\\ -E\left(\frac{{\partial }^{2}\mathcalligra{l}}{\partial p\partial \omega }\right)& -E\left(\frac{{\partial }^{2}\mathcalligra{l}}{\partial {\omega }^{2}}\right)& -E\left(\frac{{\partial }^{2}\mathcalligra{l}}{\partial \omega \partial \beta }\right)\\ -E\left(\frac{{\partial }^{2}\mathcalligra{l}}{\partial p\partial \beta }\right)& -E\left(\frac{{\partial }^{2}\mathcalligra{l}}{\partial \omega \partial \beta }\right)& -E\left(\frac{{\partial }^{2}\mathcalligra{l}}{\partial {\beta }^{2}}\right)\end{array}\right]$$

On the other hand, Fisher’s information matrix can be computed by using the approximation

$${I}_{Y}\left(\widehat{\tau }\right)=\left[\begin{array}{ccc}-{\left.\frac{{\partial }^{2}\mathcalligra{l}}{\partial {p}^{2}}\right|}_{(\widehat{p},\widehat{\omega },\widehat{\beta })}& -{\left.\frac{{\partial }^{2}\mathcalligra{l}}{\partial p\partial \omega }\right|}_{(\widehat{p},\widehat{\omega },\widehat{\beta })}& -{\left.\frac{{\partial }^{2}\mathcalligra{l}}{\partial p\partial \beta }\right|}_{(\widehat{p},\widehat{\omega },\widehat{\beta })}\\ -{\left.\frac{{\partial }^{2}\mathcalligra{l}}{\partial p\partial \omega }\right|}_{(\widehat{p},\widehat{\omega },\widehat{\beta })}& -{\left.\frac{{\partial }^{2}\mathcalligra{l}}{\partial {\omega }^{2}}\right|}_{(\widehat{p},\widehat{\omega },\widehat{\beta })}& -{\left.\frac{{\partial }^{2}\mathcalligra{l}}{\partial \omega \partial \beta}\right|}_{(\widehat{p},\widehat{\omega },\widehat{\beta })}\\ -{\left.\frac{{\partial }^{2}\mathcalligra{l}}{\partial p\partial \beta }\right|}_{(\widehat{p},\widehat{\omega },\widehat{\beta })}& -{\left.\frac{{\partial }^{2}\mathcalligra{l}}{\partial \omega \partial \beta }\right|}_{(\widehat{p},\widehat{\omega },\widehat{\beta })}& -{\left.\frac{{\partial }^{2}\mathcalligra{l}}{\partial {\beta }^{2}}\right|}_{(\widehat{p},\widehat{\omega },\widehat{\beta })}\end{array}\right]$$

where $\widehat{p}$, $\widehat{\omega }$ and $\widehat{\beta }$ are the MLEs of $p$, $\omega$ and $\beta$ respectively.

## Application

Using the proposed BDETE distribution, we examine two data sets in this section to illustrate its use. The BDETE distribution is compared to some related distributions include, the binomial geometric (BG)^12^, negative binomial-discrete Erlang-truncated exponential (NBDETE)^19^, discrete Erlang-truncated Exponential (DETE)^17^, discrete extended Erlang-truncated Exponential (DEETE)^20^, and the discrete Kumaraswamy Erlang-truncated exponential distribution (DKw_ETE)^21^ to evaluate its performance and check its goodness of fit. Both the chi-square statistic and the -log-likelihood (−log(L)) are used as evaluation tools. Two right-skewed, over-dispersed real lifetime count data sets from the cancer disease are fitted with the BDETE distribution.

The first data in Table 3, provided by Klein Moeschberger^22^ describes the death times, expressed in weeks, of 30 tongue cancer patients. This data was used by Eledum and El-Alosey^12^ to study the binomial geometric distribution. The average, variance, and skewness for this data respectively are 50.03,1945.84, and 0.972. The second data set in Table 4, released by Lawless^18^, indicates the lengths of remission in weeks for a group of 30 leukemia patients taking a specific kind of medicine. This data was utilized by Eledum and El-Alosey^12^ to assess the binomial geometric distribution. The results of the two data sets are demonstrated in Tables 5 and 6.

**Table 3:** Death times (in weeks) of patients with cancer of the tongue.

Dataset 1															
1	1	1	4	5	10	13	13	16	16	24	26	27	28	30	30
32	41	51	65	67	70	72	73	77	91	93	96	100	104	157	167

**Table 4:** Remission times, in weeks, for some leukemia patients taking a specific type of therapy.

Dataset 2														
1	1	2	4	4	6	6	6	7	8	9	9	10	12	13
14	18	19	24	26	29	31	42	45	50	57	60	71	85	91

**Table 5:** Parameters estimates, −log (L), k-s test value and p-value for the selected distributions of the tongue cancer patient’s data set.

Distribution	Distribution’s parameters			−Log(L)	k-s	p-value
BG	$\widehat{p}=0.1236$	$\widehat{\theta }=0.00246$	–	157.5224	23.69	0.5935
NB	$\widehat{p}=0.01965$	$\widehat{r}=1.0025$	–	157.5224	23.71	0.5922
DETE	$\widehat{p}=0.4672$	$\widehat{\beta }=0.02600$	–	157.5224	23.69	0.5933
DEETE	$\widehat{\alpha }=0.999$	$\widehat{p}=0.98476$	$\widehat{\beta }=1.289$	157.5224	23.69	0.5938
DKw_ETE	$\widehat{\alpha }=0.389$ $\widehat{\theta }=0.0922$	$\widehat{p}=0.3754$	$\widehat{\beta }=0.1997$	158.1346	23.14	0.5904
BDETE (proposed)	$\widehat{p}=0.54021$	$\widehat{\omega }=0.2856$	$\widehat{\beta }=0.0858$	157.487	23.12	0.5960

**Table 6:** Parameters estimates, −log (L), k-s test value and p-value for the selected distributions of the leukemia patient’s data set.

Distribution	Distribution’s parameters			−Log(L)	k-s	p-value
BG	$\widehat{p}=0.1128$	$\widehat{\theta }=0.00443$	–	127.54	33.05	0.1030
NB	$\widehat{p}=0.03907$	$\widehat{r}=1.02983$	–	127.54	33.42	0.0954
DETE	$\widehat{p}=0.4595$	$\widehat{\beta }=0.0497$	–	127.55	33.07	0.1030
DEETE	$\widehat{\alpha }=1.582$	$\widehat{p}=0.9753$	$\widehat{\beta }=1.608$	127.55	33.91	0.0863
DKw_ETE	$\widehat{\alpha }=1.471$ $\widehat{\theta }=0.1169$	$\widehat{p}=0.0042$	$\widehat{\beta }=0.0648$	127.20	38.12	0.0337
BDETE (proposed)	$\widehat{p}=0.5394$	$\widehat{\omega }=0.2728$	$\widehat{\beta }=0.0162$	127.24	33.01	0.1038

From the results in Table 5, we can see that the suggested BDETE distribution has the smallest number for −logL (157.487) compared to the other similar distributions (the smaller, the better). On the other hand, this value, along with the value of the ${\chi }^{2}$ statistic (23.12) and its associated p-value (0.5960), shows that the suggested BDETE distribution is the best model to fit the tongue cancer patient's data set. Since this is the case, all the studied distributions fit this data set well.

Table 6 shows that, among the comparative distributions, the proposed BDETE distribution has the least value for −logL (127.24**)**. This result, combined with the ${\chi }^{2}$ statistic value of (33.01) and the corresponding p-value of (0.1038) explains that the proposed BDETE distribution is the most appropriate model for the leukemia patient’s data set. On the other hand, all distributions that were considered fit the data well.

## Conclusion remarks

This paper developed a novel mixture of binomial distribution called the Binomial-discrete Erlang-truncated exponential distribution (BDETE), which was created by combining the binomial with the discrete Erlang-truncated exponential distribution using the probability generating function method. We look at some of the BDETE statistical properties and use the maximum likelihood method to estimate its parameters. The new compounding distribution has an increasing hazard rate function depending on the behavior of the distribution's parameters. Two real-world lifetime count data sets from the cancer disease, both of which are right-skewed and overdispersed, are fitted using the proposed BDETE distribution to evaluate its efficacy and viability. The application showed that the proposed distribution is the easiest model to fit a real lifetime count data set of cancer diseases that is right-skewed, over-dispersed, and has a decreasing probability mass function. We recommend using the proposed BDETE distribution for data modeling in applications of life-time count data from the medical field, especially in cancer diseases, based on the merits of increasing failure rate and decreasing probability mass function. In future studies, we can do another mixing of the BDETE distribution to increase the distribution flexibility.

## Data Availability

The datasets used and/or analysed during the current study available from the corresponding author on reasonable request.
